# Concomitant Intracranial Aneurysm Clipping and Meningioma Resection: Surgical Strategy and Considerations

**DOI:** 10.3390/cancers17172908

**Published:** 2025-09-04

**Authors:** Oday Atallah, Khadeja Alrefaie, Amr Badary

**Affiliations:** 1 Department of Neurosurgery, Evangelic Hospital Oldenburg, Carl Von Ossietzky University Oldenburg, 26111 Oldenburg, Germany; 2 Medical School Hannover, 30625 Hannover, Germany; 3 Kuwait Institute for Medical Specialization, Sulibekhat 15503, Kuwait; 19200612@rcsi.com; 4 Department of Neurosurgery, Medical University Lausitz, 03048 Cottbus, Germany; a.badary@mul-ct.de

**Keywords:** aneurysm, intracranial, tumor, meningioma, clipping

## Abstract

Objective: The simultaneous occurrence of brain aneurysms and brain tumors creates a complicated clinical scenario requiring extensive surgical planning. The study seeks to illuminate the safety, effectiveness, and prospective advantages of combination surgical intervention. Materials and Methods: We analyzed 10 patients concurrently diagnosed with both cerebral aneurysm and meningioma who received simultaneous microsurgical intervention. Clinical and demographic data, encompassing lesion characteristics, surgical specifics, complications, and outcomes, was extracted. The Karnofsky Performance Status Scale (KPS) was utilized to assess functional status preoperatively, after discharge, and during the final follow-up. Descriptive statistics and visualizations were produced via Python tools and libraries. Results: The predominant demographic was female (90%), with a median surgical age of 58 years. All patients presented with WHO Grade 1 meningiomas and predominantly incidental aneurysms, typically situated in the middle cerebral artery. At a median follow-up of 27 months, functional results demonstrated improvement, with 10% of patients reporting tumor recurrence or necessitating resurgery, while the total death rate was 20%. Conclusion: The simultaneous occurrence of intracranial aneurysms and brain tumors is infrequent. This study demonstrates that simultaneous aneurysm clipping and tumor resection with the same method is both viable and secure. A comprehensive evaluation of the patient and an extensively devised surgical plan are essential for the effective management of these complex cases.

## Simple Summary

Brain aneurysms and brain tumors are two very different problems, but in rare cases, they can occur in the same person at the same time. A brain aneurysm is a weak spot in a blood vessel that can burst and cause life-threatening bleeding. A meningioma is a usually benign tumor that grows on the coverings of the brain. When both are present, surgery becomes especially complicated. This study looked at ten patients who had both a brain aneurysm and a meningioma and were treated with one surgery to fix both problems. The team used careful planning and modern surgical techniques to safely treat both conditions during the same operation. Most of the aneurysms had not ruptured, and all tumors were of a slow-growing type. The results were promising: there were no surgical complications like bleeding during the operation, and most patients improved or stayed stable in their ability to live independently. This combined approach may help some patients avoid multiple surgeries and recover faster. The study shows the importance of careful preparation and teamwork in tackling rare but serious combinations of brain conditions.

## 1. Introduction

The concurrent occurrence of intracranial aneurysms and brain tumors is a rare and intricate clinical scenario that presents challenges to neurosurgeons for surgical planning and intervention [1,2,3]. Previously deemed coincidental, increasing evidence now indicates a possibly non-random correlation between these two entities, with recent studies documenting an occurrence of concurrent intracranial aneurysms in meningioma patients between 0.13% and 1.17%—a prevalence that surpasses what would be anticipated by mere chance [4,5,6]. This has generated interest in clarifying the processes underpinning their coexistence, encompassing tumor-induced hemodynamic alterations, hormonal influences, and the direct or indirect effects of the tumor on cerebral vasculature [7,8,9,10].

This clinical scenario necessitates a thorough and extensively devised treatment strategy, as both the positive and negative aspects of addressing each condition must be judiciously evaluated about the potential risks and interactions of concurrent surgical interventions [11,12]. The aim of concurrently clipping aneurysms and resecting brain tumors is to eradicate the immediate risk of hemorrhage while effectively excising tumor tissue within a single surgical intervention. The presence of two distinct types of entities necessitates cautious and comprehensive planning before surgery, the application of contemporary techniques during the treatment, and an in-depth understanding of cerebrovascular and neuro-oncological principles [13].

The decision-making process is often influenced by factors including the tumor’s size, location, and characteristics, along with the aneurysm’s type, location, and rupture risk. Moreover, it is essential to consider elements such as the timing of intervention, the patient’s health status, and the sequence in which each pathology is treated to optimize patient outcomes [11].

We discuss our institutional experience with ten consecutive patients who had simultaneous microsurgical clipping of cerebral aneurysms and removal of meningiomas. This work seeks to enhance the ongoing discourse by assessing the safety, practicality, and ethical implications of this intricate yet increasingly pertinent neurosurgical approach.

## 2. Methods

### 2.1. Study Design and Patient Selection

This retrospective cohort study included all patients diagnosed with coexistent intracranial aneurysm and meningioma who underwent simultaneous microsurgical treatment at our institution. Inclusion criteria were (1) radiologically or intraoperatively confirmed diagnosis of both intracranial aneurysm and meningioma; (2) surgical intervention for both lesions during the same operative session; and (3) availability of complete clinical, radiological, and outcome data. Patients with staged procedures or incomplete records were excluded. Only patients in whom both lesions were deemed accessible through a single craniotomy and whose clinical condition permitted combined surgery were included. Cases selected for staged or endovascular-first management were excluded. Selection was based on multidisciplinary review, anatomical proximity of lesions, anticipated brain shift, and patient comorbidity profile. Simultaneous management was favored when both pathologies could be safely addressed in one operative corridor with acceptable risk (flow diagram in supplementary material).

### 2.2. Data Collection

Clinical and demographic data were extracted from electronic medical records and operative reports. Variables collected included age, gender, risk factors, presenting symptoms, tumor and aneurysm characteristics (location, laterality, size, rupture status, and histopathology), surgical approach, duration of surgery, intraoperative and postoperative complications, length of hospital stay, recurrence, need for resurgery, shunt implantation, and mortality. Functional outcomes were assessed using the Karnofsky Performance Status Scale (KPS) at three time points: preoperatively, at discharge, and at last follow-up. Intraoperative monitoring included somatosensory evoked potentials and intraoperative indocyanine-green (ICG) angiography to confirm vessel patency and aneurysm occlusion. Flow probes such as the Transonic Charbel Micro-Flow probe were not routinely available in our institution but may be considered for quantitative intraoperative flow assessment in complex cases.

### 2.3. Statistical Analysis

Descriptive statistics were calculated to summarize patient characteristics, surgical details, and outcomes. Continuous variables are presented as median (range) and categorical variables as frequency (percentage). Data visualization included bar charts, pie charts, and line plots to depict distributions of clinical features, surgical outcomes, and functional trajectories. All statistical analyses and visualizations were performed using Python (version 3.11.5), with the following libraries and versions: Pandas (version 2.2.2) for data management and analysis, Matplotlib (version 3.8.4) for graphical representation of data, and NumPy (version 1.26.4) for numerical operations. No inferential statistical tests were performed due to the small sample size (*n* = 10). All analyses were conducted on anonymized data. Major complications were defined a priori as any postoperative event resulting in prolonged ICU stay, new permanent neurological deficit, need for reoperation, or death.

## 3. Results

### 3.1. Demographics and Baseline Characteristics

A total of 10 patients underwent simultaneous management for coexistent aneurysm and meningioma. The majority were female (90%), with a median age at surgery of 58 years (range: 28–86) (Table 1). The most common risk factor was hypertension (80%), followed by nicotine use (30%) and diabetes (20%). Headache (50%) and visual disturbances (40%) were the most frequent presenting symptoms.

### 3.2. Tumor and Aneurysm Characteristics

**Figure 1:**
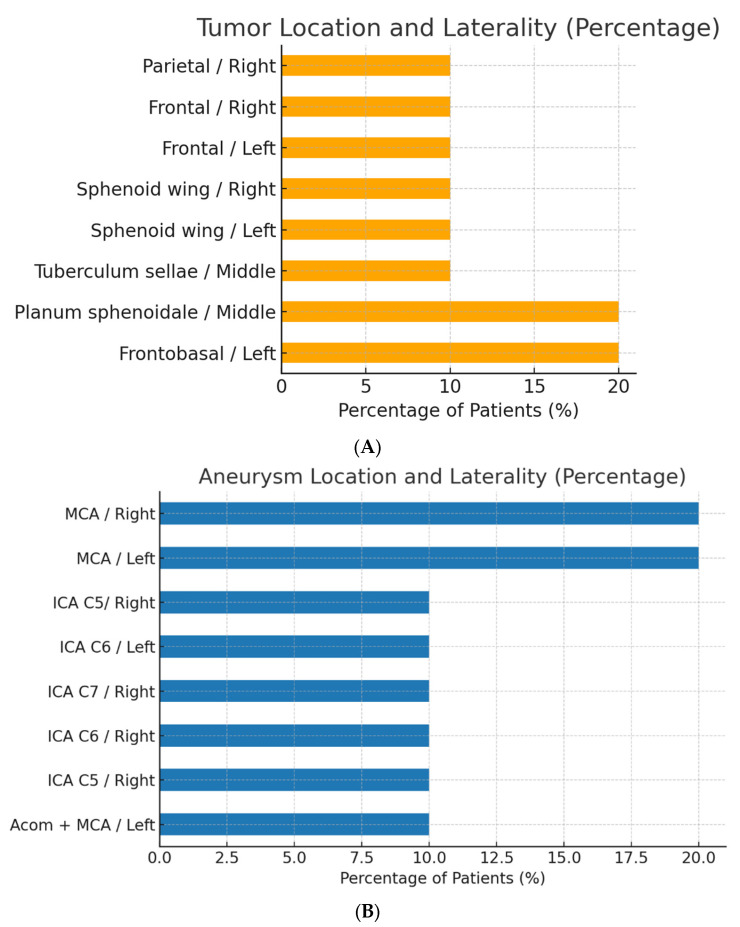
**(A) Tumor location and laterality (B) aneurysm location and laterality in patients with coexistent aneurysm and meningioma**. Horizontal bar chart illustrating the percentage of patients by tumor location and laterality. The most common tumor sites were frontobasal (left) and planum sphenoidale (middle), each accounting for 20% of cases. Other sites were each represented in 10% of patients. The middle cerebral artery (MCA) was the most common site, with equal distribution between left and right sides (20% each), followed by various segments of the anterior circulation.

**Figure 2:**
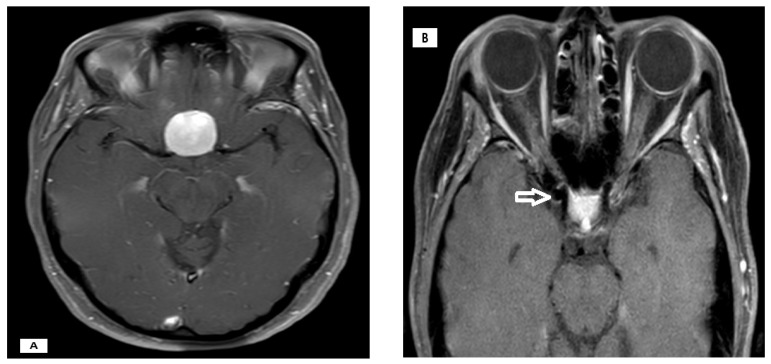
A 41-year-old female patient underwent cranial MRI as part of the diagnostic evaluation for visual disturbances. Imaging demonstrated a meningioma of the tuberculum sellae (**A**) and incidentally revealed a paraclinoid aneurysm of the internal carotid artery (as indicated by arrow) (**B**). The meningioma was resected, and in the same surgery, the aneurysm was treated by microsurgical clipping.

**Figure 3:**
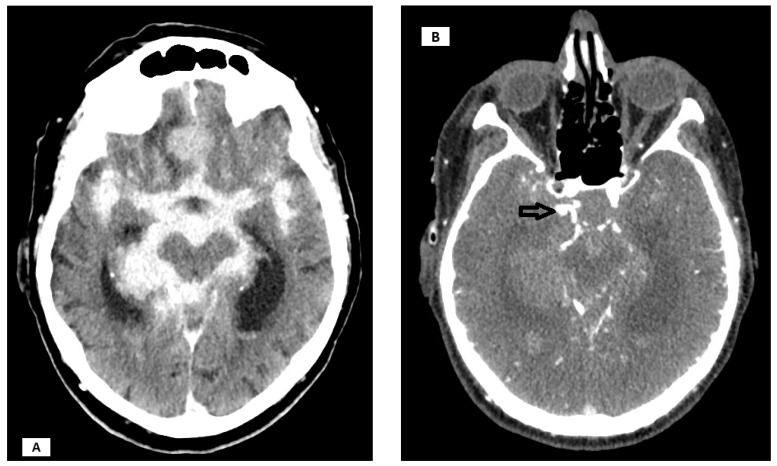
An 86-year-old female patient was admitted with an aneurysmal subarachnoid hemorrhage caused by rupture of an aneurysm located at the C7 segment of the internal carotid artery (as indicated by arrow). Cranial CT incidentally demonstrated a meningioma of the planum sphenoidale (**A**,**B**). The aneurysm was treated by microsurgical clipping, and in the same operative session, gross total resection of the meningioma was performed.

**Figure 4:**
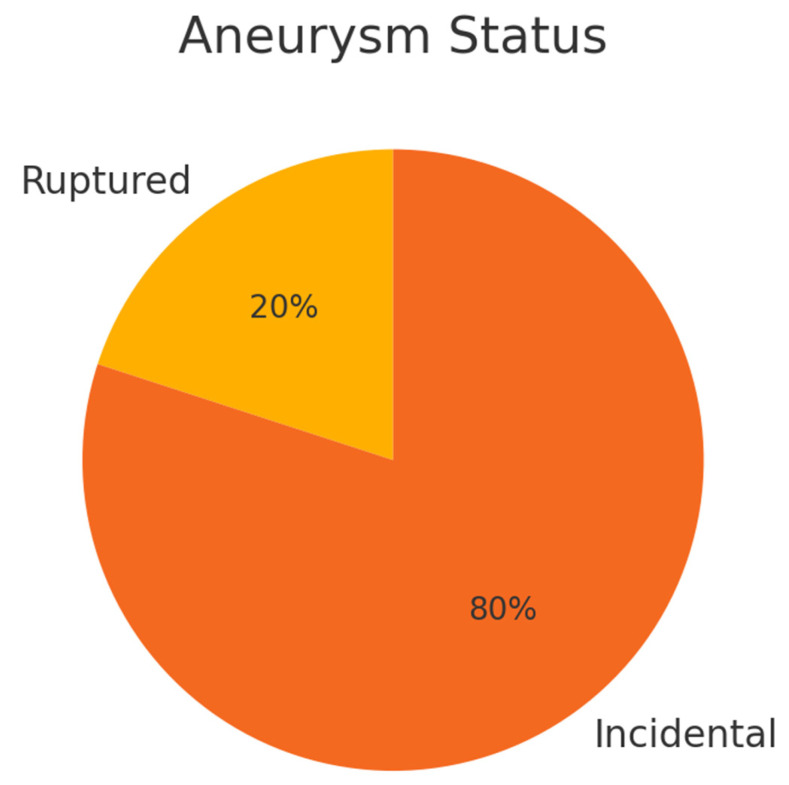
**Aneurysm status at presentation.** Pie chart depicting the distribution of aneurysm status among the cohort (*n* = 10). The majority of aneurysms were discovered incidentally (80%), while 20% presented as ruptured.

Tumors were most commonly located at the frontobasal/left (20%) and planum sphenoidale/middle (20%) regions, with other sites each accounting for 10% (Figure 1). All tumors were histopathologically classified as WHO Grade 1 meningioma. Aneurysms were most frequently located at the middle cerebral artery (MCA), left (20%) and right (20%), with other locations (including the anterior communicating artery [Acom] and various segments of the internal carotid artery [ICA]) each present in 10% of patients (Figure 2 and Figure 3). The median largest aneurysm size was 5.5 mm (range: 3–9 mm). Most aneurysms were incidental (80%), with 20% presenting as ruptured (Figure 4).

### 3.3. Surgical Details and Outcomes

The principal finding of our series is the favorable safety profile in carefully selected patients associated with the simultaneous surgical treatment of both pathologies. All patients underwent single-stage microsurgical aneurysm clipping and tumor resection, most commonly via an ipsilateral (right) pterional approach. The median duration of surgery was 200 min (range: 145–645). Complete aneurysm occlusion was achieved in all cases (100%), with no remnants observed (Table 1). There were no cases of major perioperative complications such as procedure-related bleeding or intraprocedural rupture; however, there were 20% of cases who had hydrocephalus and vasospasm, and those were the two patients that had ruptured aneurysms (subarachnoid hemorrhage).

### 3.4. Postoperative Course and Follow-Up

**Figure 5:**
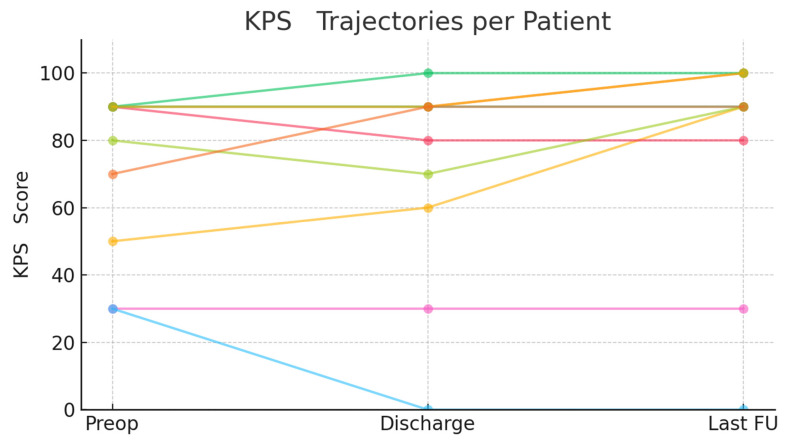
**Functional outcomes (KPS) over time.** Line plot displaying individual patient trajectories for the Karnofsky Performance Status Scale (KPS) at three time points: preoperative, discharge, and last follow-up. Most patients maintained or improved their functional status following simultaneous surgical intervention.

The median hospital stay was 15 days (range: 7–40). There were no cases of postoperative rebleeding. Recurrence and the need for resurgery for tumors occurred in 10% of cases each. The median KPS was 85 preoperatively (range: 30–90), 85 at discharge (range: 0–100), and 90 at last follow-up (range: 0–100) (Figure 5). The median follow-up duration was 27 months (range: 12–36), with an overall mortality of 20%. Of the two deaths (20%), both occurred in patients with ruptured aneurysms presenting with diffuse SAH. One patient died within 22 days from delayed cerebral ischemia; the second died 33 months later from medical complications. No deaths were directly attributable to intraoperative events. Vasospasm and hydrocephalus were observed only in the ruptured subgroup and managed per standard SAH protocols. These events were considered serious complications, and their confinement to the SAH subgroup reflects the inherent severity of rupture rather than the surgical strategy per se.

## 4. Discussion

Intracranial aneurysms and brain tumors represent major concerns in neurovascular and neurooncological surgery due to their potentially devastating outcomes and the complexities involved in their diagnosis. An intracranial aneurysm is a localized dilatation or bulging of a cerebral blood artery, typically resulting from a weakening in the arterial wall [14,15,16]. These aneurysms may be asymptomatic but pose a danger of rupture, resulting in subarachnoid hemorrhage [17,18,19]. The anterior communicating artery is the most common site for aneurysm formation, followed by the posterior communicating artery and the middle cerebral artery, mostly due to hemodynamic stress in these areas of the Circle of Willis [20,21,22,23].

On the other hand, brain tumors comprise a diverse array of neoplastic formations within the cranial cavity, differing in histological classification, growth characteristics, and prognosis. Meningiomas are the most prevalent primary brain tumors, constituting almost one-third of all intracranial neoplasms [24,25,26,27]. Those originating from the arachnoid cap cells of the meninges are typically benign and exhibit sluggish growth; nonetheless, their location and size may result in considerable neurological impairments due to compression of surrounding brain regions [28,29].

Recent literature estimates that the incidence of concurrent intracranial aneurysms in patients with meningiomas ranges from 0.13% to 1.17%, higher than chance alone would predict. [6] Various mechanisms have been hypothesized, including tumor-induced alterations in regional hemodynamics, hormonal influences (notably in female-predominant cohorts like ours), and chronic irritation of arterial walls by the tumor mass.

Given the female predominance and invasive nature of the procedures, preoperative screening for coagulation disorders may be prudent. Although not performed routinely in our series, future protocols may benefit from incorporating screening for occult platelet function defects or von Willebrand disease in patients without obvious risk factors, as recently proposed by Wagner et al. (2023) [30]. Rabello et al. (2024) systematically reviewed the literature and highlighted several hypothesized pathways of coexistent meningiomas and aneurysms [31]. Mechanical changes induced by tumor progression (such as compression or traction on cerebral vessels) may result in regional alterations of blood flow and increased hemodynamic stress, thereby promoting aneurysm formation [31]. Hormonal factors, particularly in tumors associated with growth hormone production, may also contribute by causing systemic hypertension and vascular wall weakness [31]. Additionally, direct infiltration of the arterial wall by tumor cells, as well as chronic irritation or inflammatory changes induced by the tumor mass, have been suggested as possible mechanisms, though this is more clearly established for some tumor types than others. However, the true nature of this association remains incompletely understood and is an important area for future research. Our analysis of 10 consecutive patients who underwent single-stage microsurgical management for both lesions provides important insights into the safety, feasibility, and ethical dimensions of this complex intervention.

Our findings support prior studies suggesting that, in carefully selected patients, simultaneous surgical management is both feasible and safe, provided that both the aneurysm and tumor are accessible through a single craniotomy and that the patient’s overall condition is suitable for a potentially prolonged operation. This is supported by Aljuboori et al., who reported the case of a patient with a dural-based temporal brain tumor and an ipsilateral unruptured anterior cerebral artery (A1 segment) aneurysm [1]. The surgical team performed a single-stage procedure through a left frontotemporal craniotomy, enabling both gross total resection of the tumor and successful clipping of the aneurysm via the same operative corridor [1]. The patient recovered well, with only a transient neurological deficit that resolved prior to discharge [1]. The authors concluded that, in appropriately selected cases where both lesions are accessible via a single approach, contemporaneous surgical management is both reasonable and effective [1].

Importantly, single-stage intervention minimizes the risks associated with multiple anesthetic exposures and the potential hemodynamic instability of staged procedures, especially in cases where the presence of an unaddressed aneurysm could increase the risk of intraoperative rupture during subsequent tumor resection, or vice versa. This concept is further exemplified by Zhou et al. (2024), who reported a rare and complex case involving a 53-year-old man presenting with SAH due to a ruptured left posterior communicating artery (PcomA) aneurysm [32]. Preoperative imaging also revealed a contralateral, unruptured right clinoid aneurysm, but a coexisting meningioma of the left anterior clinoid process was only identified intraoperatively due to its small size and lack of radiological prominence [32]. The surgical team elected to perform an urgent left frontotemporal craniotomy [32]. In a single-stage procedure, they completely resected the meningioma and successfully clipped both the ruptured left PcomA aneurysm and the unruptured right clinoid aneurysm using the same surgical corridor [32]. The patient tolerated the prolonged, complex operation well and experienced no new postoperative neurological deficits [32]. Follow-up imaging confirmed total resection of the meningioma and secure obliteration of both aneurysms. The authors emphasized that excellent microsurgical technique and thorough intraoperative assessment are critical for such challenging cases and concluded that when lesions are adjacent or accessible through one approach, simultaneous surgical management can be both safe and effective [32]. Intraoperative blood-flow monitoring, such as with Transonic Intracranial Charbel Micro-Flow probes, has been reported to aid in the qualitative and quantitative assessment of vessel flow before and after clipping, particularly in complex aneurysms or when temporary clipping is applied [33,34]. In our series, Charbel probes were not routinely used, largely due to institutional availability and the relatively small size and straightforward morphology of the aneurysms encountered. However, in future cases, especially involving posterior circulation or giant aneurysms, the use of such probes could offer additional safety by confirming adequate perfusion and clip placement. The management approach differed between ruptured and unruptured aneurysms in our series. For ruptured aneurysms (20% of cases), the urgency of addressing the source of hemorrhage dictated surgical timing and prioritization. In these cases, the aneurysm was secured first, followed by tumor resection in the same session only if patient stability permitted. Additional measures such as temporary clipping, meticulous brain relaxation, and vasospasm prophylaxis were employed. For unruptured aneurysms, the surgical sequence was more flexible, and tumor resection was often initiated first to decompress the operative corridor, followed by aneurysm clipping. The decision was tailored based on lesion location, vascular anatomy, and anticipated brain shift. The feasibility and safety of simultaneous surgical management in patients with multiple intracranial lesions is further demonstrated by Wei et al. (2022) [11]. In their case report, a 38-year-old man with three meningiomas and three unruptured aneurysms underwent a single-stage surgery in which two meningiomas were resected and all three aneurysms were clipped through a single craniotomy [11]. The patient had an excellent postoperative recovery with only one minor, self-limited seizure and remained symptom-free at follow-up. The authors concluded that when aneurysms are located within the operative field of a planned craniotomy for tumor resection, simultaneous management is both feasible and safe in appropriately selected patients [11]. Additionally, a recent systematic review by Abou-Mrad et al. (2024) [35] evaluated 115 patients with concurrent meningioma and intracranial aneurysm, finding that 34% of unruptured cases underwent single-stage surgery for both pathologies via a single craniotomy. The review concluded that, when the aneurysm and tumor are in close proximity and accessible from the same operative approach, simultaneous surgical management is a safe and effective strategy for appropriately selected patients [35]. The authors propose that the decision should be individualized based on the anatomical relationship of the lesions, risk of aneurysm rupture, and patient-specific factors, but support single-stage management in suitable cases [35].

The median operative time (200 min) found in our cohort reflects the technical demands of this approach, but outcomes in terms of functional status (as measured by KPS) were excellent, with median scores improving from 85 preoperatively to 90 at last follow-up. In unruptured cases, tumor decompression was generally performed first to widen the corridor, followed by aneurysm dissection and clipping. In ruptured cases, aneurysm security took priority, with tumor resection added if patient stability permitted. Temporary clips were selectively applied, and vasospasm prophylaxis (nimodipine irrigation, euvolemia) was employed in SAH cases. Our series also emphasizes the need for robust preoperative imaging and planning: in 20% of cases, patients harbored multiple aneurysms, and all tumors were classified as WHO Grade I, suggesting that a benign tumor phenotype and accessible aneurysm location (predominantly MCA territory) are optimal candidates for this approach.

However, the surgical strategy must remain individualized. Certain anatomical configurations (such as deep-seated or posterior circulation aneurysms or high-grade/atypical meningiomas) may still favor a staged approach. While all aneurysms in our series were located in the anterior circulation, the management of unruptured posterior-circulation aneurysms requires special consideration. Given the deep location and narrow surgical corridors, many such aneurysms are more safely and effectively treated using endovascular techniques such as coiling or flow-diversion [36]. In cases where a posterior-circulation aneurysm coexists with a surgically approachable meningioma, a hybrid approach (endovascular treatment of the aneurysm followed by surgical tumor resection) may be the most appropriate strategy. We recommend multidisciplinary case discussions and individualized treatment planning for these scenarios.

**Figure 6:**
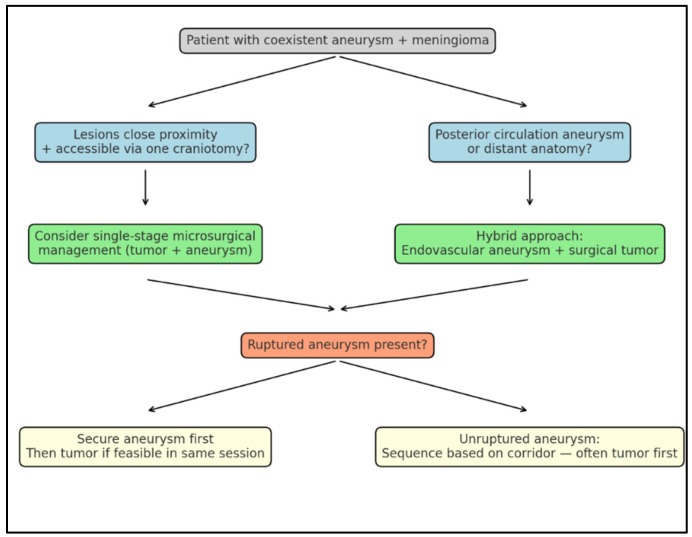
**A simplified decision aid diagram to guide surgical strategy in patients with coexistent meningioma and aneurysm.** If both lesions are in close proximity and accessible via one craniotomy, and patient’s condition allows, consider single-stage microsurgical management. If the aneurysm is in the posterior circulation or anatomically distant, consider a staged or endovascular-first approach. If the aneurysm is ruptured, prioritize securing the aneurysm, with tumor resection only if safely feasible in the same session. If the tumor is symptomatic or rapidly growing, consider earlier tumor resection, with aneurysm clipping concurrently if low-risk.

To translate our institutional experience into practice, we propose a compact decision aid (Figure 6) outlining when simultaneous surgery is preferable versus staged or hybrid management.

A second, less frequently addressed dimension is the ethical complexity that arises when a tumor is identified intraoperatively—sometimes unexpectedly—during planned aneurysm surgery, or vice versa. This scenario poses significant challenges: the patient has typically consented to one procedure, and the intraoperative discovery of a second, surgically approachable pathology compels the surgical team to make real-time decisions about whether to proceed.

From an ethical standpoint, this situation involves a balance between beneficence (acting in the patient’s best interest), respect for patient autonomy, and non-maleficence (avoiding harm). On the one hand, addressing both lesions in a single operation may spare the patient a second high-risk craniotomy, reduce healthcare costs, and shorten total recovery time. On the other hand, it may extend operative time beyond what was consented, potentially increasing the risk of complications, and may subject the patient to a procedure they did not fully anticipate.

The principle of informed consent is paramount. Ideally, the possibility of intraoperative discovery of a second lesion should be discussed preoperatively—especially in patients with radiologically ambiguous findings or a high index of suspicion for concurrent pathology—so that consent can be obtained for both planned and possible extended interventions. In cases where this is not feasible, the surgical team should be guided by the best available evidence, intraoperative assessment, and, when possible, intraoperative consultation with family or legal proxies.

This emphasizes the need for structured protocols and ethical frameworks in neurosurgical practice, including the use of intraoperative imaging, multidisciplinary discussion, and, when possible, “real-time” communication with patient surrogates. It is also critical to document all intraoperative findings and decision-making processes thoroughly, ensuring transparency and accountability.

**Limitations**: Our series is limited by its retrospective design, modest sample size, and restriction to WHO Grade I meningiomas and accessible aneurysms. Generalizability to higher-grade tumors, posterior circulation aneurysms, or patients with major comorbidities is therefore limited. Nevertheless, the detailed reporting of complications, outcomes, and ethical considerations adds meaningfully to the growing literature on this complex topic. We advocate for the establishment of multi-institutional registries and prospective studies to further define best practices and patient selection criteria.

## 5. Conclusions

In summary, simultaneous microsurgical management of coexistent intracranial aneurysm and meningioma can be performed safely and effectively in selected patients, with functional outcomes at least equivalent to staged approaches. Preoperative planning, intraoperative vigilance, and clear, ethically sound communication with patients and families are essential to optimizing outcomes and maintaining trust in the neurosurgical enterprise.

## Figures and Tables

**Table 1:** Demographics, risk factors, symptoms, tumor and aneurysm location and laterality, aneurysm characteristics, surgical details and outcome, complications, postoperative course and follow-up.

	Variable	Value
**Demographics**	**Number of Patients**	10
	**Female (%)**	90.0
	**Median age (range)**	58.0 (28–86)
**Risk Factor**	**Hypertension (%)**	80.0
	**Nicotine (%)**	30.0
	**Diabetes (%)**	20.0
**Most Common Presenting Symptoms**	**Headache (%)**	50.0
	**Visual disturbances (%)**	40.0
	**Ataxia (%)**	30.0
**Tumor Location and Laterality**	**Frontobasal/left (%)**	20.0
	**Planum sphenoidale/middle (%)**	20.0
	**Other locations (each 10%)**	Tuberculum sellae/middle, sphenoid wing/left, sphenoid wing/right, frontal/left, parietal/right, frontal/right
	**Tumor histopathology**	WHO grade 1 meningioma (100%)
**Aneurysm Location and Laterality**	**MCA/left (%)**	20.0
	**MCA/right (%)**	20.0
	**Other locations (each 10%)**	Acom + MCA/left, ICA C5/right, ICA C6/right, ICA C7/right, ICA C6/left, ICA C5/right
**Aneurysm Characteristics**	**Aneurysm size (mm) median (range)**	5.5 (3–9)
	**Ruptured aneurysms (*n*, %)**	2 (20.0%)
	**Incidental aneurysms (*n*, %)**	8 (80.0%)
**Surgical Details and Outcome**	**Single-stage surgery (microsurgical) (%)**	100.0
	**Most common surgical approach**	Pterional right
	**Duration of surgery median (range), minutes**	200 (145–645)
	**Complete occlusion rate (%)**	100.0
	**Remnant after surgery (%)**	0.0
**Complications**	**Hydrocephalus (%)**	20.0
	**Vasospasm (%)**	20.0
	**Bleeding (%)**	0.0
	**Intraprocedural rupture (%)**	0.0
	**Shunt implantation (%)**	10.0
**Postoperative Course and Follow-up**	**Median hospital stay (days) (range)**	15.0 (7–40)
	**Rebleed (%)**	0.0
	**Recurrence (%)**	10.0
	**Resurgery for tumor (%)**	10.0
	**KPS preop median (range)**	85.0 (30–90)
	**KPS at discharge median (range)**	85.0 (0–100)
	**KPS at last FU median (range)**	90.0 (0–100)
	**Median follow-up (months) (range)**	27.0 (12–36)
	**Mortality (%)**	20.0

## Data Availability

The data presented in the following study are available from the corresponding authors upon request.

## Supplementary Materials

The following supporting information can be downloaded at https://www.mdpi.com/article/10.3390/cancers17172908/s1, Figure S1: Patient Selection Process.
