# Supplementary figures and images for: Concomitant Intracranial Aneurysm Clipping and Meningioma Resection: Surgical Strategy and Considerations
Source: Cancers (Basel). 2025 Sep 4;17(17):2908. doi: 10.3390/cancers17172908 (PMC12428454; doi:10.3390/cancers17172908)

## Supplementary Material

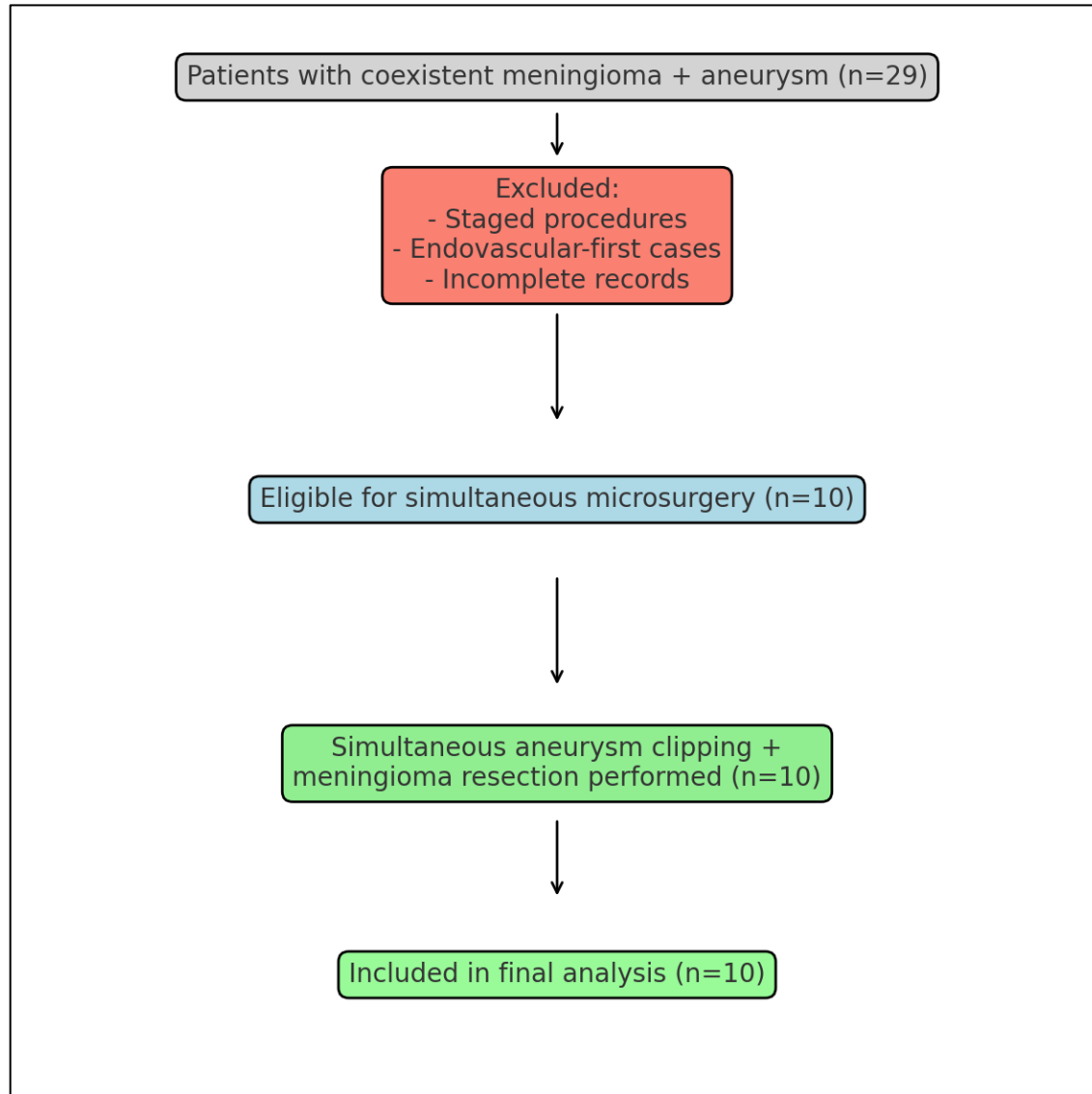

**Figure S1.** Patient selection process.

Supplement: Supplementary file 1 [file cancers-17-02908-s001.zip › cancers-3799217-supplementary.pdf]
